# The involvement of human organic anion transporting polypeptides (OATPs) in drug-herb/food interactions

**DOI:** 10.1186/s13020-020-00351-9

**Published:** 2020-07-09

**Authors:** Youmna Ali, Tahiatul Shams, Ke Wang, Zhengqi Cheng, Yue Li, Wenying Shu, Xiaofeng Bao, Ling Zhu, Michael Murray, Fanfan Zhou

**Affiliations:** 1 Faculty of Medicine and Health, Sydney Pharmacy School, The University of Sydney, Camperdown, NSW 2006 Australia; 2 Key Laboratory of Nuclear Medicine, Ministry of Health, Jiangsu Key Laboratory of Molecular Nuclear Medicine, Jiangsu Institute of Nuclear Medicine, Wuxi, Jiangsu China; 3 Department of Pharmacy, Affiliated Cancer Hospital & Institute of Guangzhou Medical University, Guangzhou, Guangdong Province 511400 China; 4 School of Pharmacy, Nantong University, Nantong, Jiangsu Province 226019 China; 5 The University of Sydney, Save Sight Institute, Sydney, NSW 2000 Australia; 6 Faculty of Medicine and Health, Discipline of Pharmacology, The University of Sydney, Camperdown, NSW 2006 Australia

**Keywords:** Organic anion transporters, Drug-herb interaction, Drug-food interaction, Therapeutic toxicity

## Abstract

Organic anion transporting polypeptides (OATPs) are important transporter proteins that are expressed at the plasma membrane of cells, where they mediate the influx of endogenous and exogenous substances including hormones, natural compounds and many clinically important drugs. OATP1A2, OATP2B1, OATP1B1 and OATP1B3 are the most important OATP isoforms and influence the pharmacokinetic performance of drugs. These OATPs are highly expressed in the kidney, intestine and liver, where they determine the distribution of drugs to these tissues. Herbal medicines are increasingly popular for their potential health benefits. Humans are also exposed to many natural compounds in fruits, vegetables and other food sources. In consequence, the consumption of herbal medicines or food sources together with a range of important drugs can result in drug-herb/food interactions via competing specific OATPs. Such interactions may lead to adverse clinical outcomes and unexpected toxicities of drug therapies. This review summarises the drug-herb/food interactions of drugs and chemicals that are present in herbal medicines and/or food in relation to human OATPs. This information can contribute to improving clinical outcomes and avoiding unexpected toxicities of drug therapies in patients.

## Background

Solute carrier transporters (SLCs) are transmembrane proteins that mediate the cellular uptake of endogenous and exogenous substances. The SLC superfamily consists of over 300 isoforms, many of which are localised at the plasma membrane of cells [1]. Organic anion transporter polypeptides (OATPs), encoded by the *SLCO* genes, are one of the most important SLC subfamilies that contribute to drug and endobiotic uptake into cells [2]. There are 11 known OATP isoforms, which are widely distributed across tissues such as the kidney, liver, intestine and brain [3].

Most OATP substrates are large hydrophobic anions with molecular weight > 350 Da. Some endogenous chemicals of physiological importance are substrates including steroids, bile acids, prostaglandins and thyroid hormones. OATPs also have important roles in the absorption, distribution and elimination of drugs such as anti-cancer agents, antibiotics, antivirals and statins [4]. Therefore, OATP function strongly influences the pharmacokinetics and pharmacodynamics of drugs and, in turn, their therapeutic outcomes. The dysfunction of OATPs may lead to suboptimal drug treatment and/or unexpected toxicities.

The transport mechanism of OATPs remains largely unclear. However, studies to date have suggested that substrate transport by OATPs is ATP and sodium independent. OATPs appear to act as electroneutral exchangers that couple substrate uptake to the cellular efflux of a counterion, such as bicarbonate, glutathione, conjugated glutathione or glutamate [4, 5].

### General information about the major OATP isoforms

OATP1A2, OATP1B1, OATP1B3 and OATP2B1 have been well studied for their important roles in the kidney and liver. These isoforms have been extensively investigated in relation to a wide range of drugs.

OATP1A2 is the first identified and best characterized human OATP isoform. This transporter is located at the apical membrane of the distal nephron, where it mediates urinary drug secretion and reabsorption. It is also expressed in hepatic cholangiocytes, where it modulates the secretion of molecules into the bile duct. It has also been discovered in the brain endothelial cells that comprise the blood–brain barrier, at the apical membrane of intestinal enterocytes and at the apical membrane of retinal epithelial cells [6–8]. OATP1A2 is known to transport a broad range of clinically important drugs like methotrexate, imatinib and fexofenadine. Overall, it plays an important role in the absorption, distribution and elimination of various drugs and substances [3, 5].

OATP1B1 is selectively expressed at the basolateral membrane of hepatocytes [5]. It can transport many widely used drugs such as statins and anti-viral agents, as well as endogenous substances like estrone-3-sulfate (ES) and bilirubin.

Like OATP1B1, OATP1B3 is also specifically located at the basolateral membrane of hepatocytes, but it is primarily expressed around the central vein [3]. The substrate specificities of OATP1B1 and OATP1B3 somewhat overlap [4]. However, OATP1B3 has relatively less impact on drug pharmacokinetics than OATP1B1 considering there are relatively less reports on the influence of OATP1B3 on drug performance [3].

OATP2B1 is highly expressed at the basolateral membrane of hepatocytes where it modulates drug influx into the liver. It is also located at the apical membrane of the enterocytes, where it influences drug absorption. In the kidney, it mediates the secretion of molecules into and/or reabsorption from urine. It has also been identified in the placental syncytiotrophoblasts, skeletal muscle and endothelial cells of the blood–brain barrier [6, 9–12]. Many clinically important drugs such as atorvastatin, pravastatin and glibenclamide, as well as the endogenous compounds like ES, bile acids, pregnenolone sulphate and prostaglandins, have been found to be OATP2B1 substrates [5, 13].

### OATP regulations in health and disease

OATP transporters can be regulated at the transcriptional and post- translational levels by a number of signalling pathways and kinases [1, 2]. For instance, kinases like protein kinase C (PKC) modulate the trafficking of OATP1A2, OATP1B1, OATP1B3 and OATP2B1 between the plasma membrane and intracellular compartments [13–15]. Similarly, casein kinase-2 also influences the expression and function of OATP1A2 by altering its subcellular trafficking to the plasma membrane [1].

Literature reports have shown that the expression and/or function of OATPs is altered in cancer and other disease states. OATPs may be novel tumour biomarkers and may influence the progression of hormone-dependent cancers [16]. For example, OATP1A2 protein expression is almost tenfold higher in breast cancer patients than that in normal subjects [17, 18]. This could be clinically significant, because OATP1A2 increases the uptake of ES, the major precursor of biologically active estrogen, which may enhance tumour cell proliferation and survival. OATP1B1 is normally liver specific but is over-expressed in tumours of the colon, lung, breast, prostate, ovary and pancreas [19–22]. Similarly, OATP1B3 is found to be overexpressed in breast, prostate and colorectal cancers [21, 23]. An important OATP1B3 substrate is testosterone, which has been shown to decrease the survival of patients with androgen dependent prostate cancer [24]. OATP2B1 expression is increased in breast, thyroid, glioma, prostate and testicular cancers [6, 24, 25]. This may enhance the uptake of ES into estrogen receptor (ER)-positive breast tumour cells and that of dehydroepiandrosterone into prostate cancer cells [23]. Again, OATP2B1 could promote tumorigenesis by enhancing cancer cell proliferation due to these effects [26]. Additionally, the expression of the orphan OATP transporter OATP5A1 is shown to be elevated in metastatic cancers [16]. Therefore, the altered expression of OATPs in the cancers mentioned above is closely related to cancer progression.

Abnormal OATP expression and function has also been reported in inflammatory conditions such as fibrosis, inflammatory bowel disease, cholestasis and advanced liver diseases, greatly contributing to disease progression [27]. For instance, OATP1A2 mRNA was increased in the placentas of pregnant patients with intrahepatic cholestasis [28]. OATP2B1 expression was upregulated in the patients with Crohn’s disease and ulcerative colitis [29]. Inflammatory bowel disease has been found to be associated with the increased level of OATP2B1 and OATP4A1 in the ileum and colon [29]; OATP4A1 is also reported to be upregulated in polycystic ovarian syndrome [30]. In contrast, the expression of OATP2B1 was decreased in the placentas of patients with bacterial chorioamnionitis to ~ 50% of the age-matched controls [31]. And OATP1B1 was also shown to be decreased in the livers of patients with primary sclerosing cholangitis [32] or severe viral hepatitis [27, 33]. Previous findings also suggested that cytokines such as TNF-α, IL-6, IL-1β, IFN-γ and oncostatin M, may regulate the expression of OATPs in cells [34–36].

Post-translational glycosylation of OATP1B1, 1B3 and 2B1 was decreased in the livers of patients with severe non-alcoholic fatty liver disease [37]. This might impair the subcellular trafficking of OATPs. Proteomics analysis showed that OATP2B1 was found to be increased in hepatic cirrhosis induced by hepatitis C infection, although the expression of OATP1B1 and OATP1B3 was unchanged [38]. Cholestasis also decreased the hepatic expression of OATP1A2, OATP1B1 and OATP1B3 at mRNA level [39–41]. Together, these findings provide insights into how severe liver diseases complicate the effectiveness of drug therapies by modulating OATP function and expression and thus, cellular influx of their drug substrates.

### The influence of OATP genetic polymorphisms on drug performance

Studies have shown that several OATP genetic variants are possibly associated with the pathogenesis of human diseases. For example, mutations in the *SLCO1B1* and *SLCO1B3* genes have been implicated in Rotor syndrome [42]. When both transporters are defective, bilirubin is taken up by the liver inefficiently leading to serum accumulation and jaundice [43]. There have also been reports that patients carrying specific OATP1B1 polymorphisms are at an increased risk of severe hyperbilirubinaemia [44–46]. And OATP1A2 genotypes were also found to be involved in the neurodegenerative disease progressive supranuclear palsy; while OATP2A1 polymorphisms are indicated to be associated with primary hypertrophic osteoarthropathy [4, 47].

Genetic polymorphisms of OATPs may have great impact on pharmacokinetic performance of drugs. It has been shown that the single nucleotide polymorphisms of OATP1B1 result in the altered disposition of statins [48, 49]. Systemic exposure to the anti-diabetic drug nateglinide [46] and to the HIV protease inhibitor lopinavir is also increased in these individuals [50]. OATP1A2 polymorphisms have also been found to be associated with impaired imatinib clearance [51] and OATP2B1 polymorphisms could impact on fexofenadine pharmacokinetics [52].

Sebastian et al. found that OATP5A1 is atypical as it does not transport classic OATP substrates [53]. Instead, it appears to be a determinant of cell shape, differentiation and motility. Microarray studies have shown that OATP5A1 is widely expressed in the fetal brain, prostate, skeletal muscle and thymus. Like other OATPs, defective OATP5A1 has been associated with human pathologies. Its genetic deletion appears to be related to Mesomelia synostoses syndrome, which is a congenital disease characterised by short limb and malformation [54].

### The interactions of herb/diet-derived chemicals with OATPs

Herbal and dietary supplements are currently popular in treating a wide range of health conditions. For example, silymarin, an active ingredient of milk thistle, can be used to treat intoxication caused by ingestion of death cap mushrooms [55]. St.John’s Wort is a herbal supplement that has been applied to treat depression [56]. One of the most common dietary products—green tea—is commonly adopted in weight loss programs; and despite definitive supporting evidence of efficacy, to prevent cancer and cardiovascular diseases [57]. Although complementary therapies and food are relatively safe, evidence to the contrary is increasing. In particular, some herbal preparations or food consumption may elicit clinically significant adverse reactions when co-administered with conventional medicines [58]. This problem is exacerbated by the ready availability of herbal and dietary supplements as well as the fact that people may self-medicate.

OATP1A2, 1B1, 1B3 and 2B1 contribute extensively to the disposition of drugs in humans [4]. The co-administration of drugs or other molecules that compete with specific OATP isoforms has the potential to elicit pharmacokinetic interactions [59]. During the pre-clinical phase of drug development, regulatory authorities require the evaluation of potential interactions involving major OATPs. However, herbal medicines derived from plants, fruits and vegetables widely used in many countries, are not subject to regulatory approval. Precautions are essential when herbal medicines and conventional drugs are co-administered and elicit interactions. Drug-drug interactions involving OATPs have been widely described in literature [60–62]; however, drug-herb/food interactions associated with these transporters have not been extensively reviewed so far.

As mentioned, a wide range of herbal and dietary compounds are substrates and inhibitors of OATPs [27, 63–67]. Unexpected adverse effects may occur if a conventional drug with a narrow therapeutic index competes for specific OATPs with herb/food chemicals [27]. Accordingly, a greater appreciation of potential drug-herb/food interactions involving OATPs could contribute to improved efficacy and safety of drug therapies when co-administered with herbal or food supplements. Table 1 summarises the documented interactions of commonly used herbal medicines or food-derived chemicals with human OATP transporters.

**Table 1:** Potential drug-herb/food interactions involving OATPs

Herbal or dietary product	Chemicals identified in extracts	OATP	Substrate	Effect on transporter	apparent IC_50_	K_i_	Cell model	Refs.
Bofutsushosan extract		2B1	ES	Inhibition	14 μg/ml		HEK 293	[71]
Rhei Rhizoma	EGCG ECG (+)-catechin EC EGC anthraquinones emodin	2B1	ES	Inhibition	3.7 μg/ml		HEK 293	[71]
Perillae herba extract	Scutellarin	2B1	ES	Inhibition	8.2 μg/ml		HEK 293	[71]
Scuterallia Radix	Baicalin	2B1	ES	Inhibition	11 μg/ml		HEK 293	[67]
Glyryrrhizae Radix Licorice	Glycyrrhizic acid	2B1 1B1	ES ES	Inhibition Inhibition	18 μg/ml 14.6 μM		HEK 293 HEK 293	[71] [72]
Moutan cortex	β-PGG	2B1	ES	Inhibition	31 μg/ml		HEK 293	[73]
Paeoniae Radix	β-PGG	2B1	ES	Inhibition	0.15 mg/ml		HEK 293	[71]
Tribuli Fructus Saussurea Radix Curcumae Rhizoma Tomato Buckwheat	Rutin	2B1	ES	Activation			HEK 293	[71]
		1B1	DHEAS	Activation			HeLa	[74]
Chinese skullcap	Baicalin Baicalin Baicalein	2B1	ES	Inhibition	5.6 ± 3.2 μM		HEK 293	[67]
		1B3	CCK-8		13.7 ± 3.6 μM 7.7 ± 2.4 μM		HEK 293	[67]
Horny goat weed	Icariin	2B1 1B1	ES	Inhibition	6.4 ± 1.9 μM 21.9 ± 2.0 μM		HEK 293	[27] [63]
		1B3	CCK-8		3.0 ± 1.3 μM			
Radix Astragali	Astragaloside	1B1	ES	Inhibition	25.5 μM		HEK 293	[72]
Panax ginseng	Ginsenoside Rc	1B1	ES	Inhibition	18.5 μM		HEK 293	[72]
Pyrola incarnate Fisch	2-O-galloyl hyperin	1B1	ES	Inhibition	19 μM		HEK 293	[72]
Herba Epimedii	Epimedin C	1B1	ES	Inhibition	23.5 μM		HEK 293	[72]
Milk thistle	Silymarin	1B1	Estradiol-17β-glucuronide	Inhibition	1.3 μM		HEK 293	[75]
		1B3	Estradiol-17β-glucuronide	Inhibition	2.2 μM		HEK 293	[75]
		2B1	ES	Inhibition	0.3 μM		MDCKII	[75]
Breviscapine	Scutellarin	1B1	ES	Inhibition	28.4 μM		HEK 293	[72]
		2B1	ES	Inhibition	2 μM		HEK 293	[76]
Grapefruit juice	Naringenin	2B1	ES	Inhibitor	16 μM		HEK 293	[71]
		1A2	Fexofenadine	Inhibitor	3.6 μM		HeLa	[77]
	hesperidin	2B1		Inhibitor				
		1A2	Fexofenadine	Inhibitor	2.7 μM		HeLa	[77]
Apple juice	Phlorizin	1B1	DHEAS	Inhibition			HeLa	[74]
	Quercetin	1A2	BSP	Inhibition		22 μM	HEK 293	[78]
		2B1	BSP	Inhibition		8.7 μM	HEK 293	[78]
		1B1	ES	Inhibition	20.4 μM		HEK 293	[72]
	Kaempferol	1A2	BSP	Inhibition		25.5 μM	HEK 293	[78]
		2B1	BSP	Inhibition		15.1 μM	HEK 293	[78]
Black tea	Theaflavin	2B1	ES	Inhibition		8.2 μM	HEK 293	[79]
Green tea	ECG EGCG ECG EGCG ECG EGCG ECG EGCG	1B1	ES	Inhibition	58.6 μM 7.8 μM		CHO	[65]
		2B1	ES	Inhibition	35.9 μM 101 μM		CHO	[65]
		1A2	ES	Inhibition	10.2 μM 54.8 μM	10.4 μM 18.8 μM	HEK 293	[65]
		1B3	ES	Activation		34.1 μM 13.2 μM	CHO	[65]
Pomegranate	Ursolic acid	2B1	ES	Inhibition	11.0 ± 5.0 μM		HEK 293	[64]
		1B1	ES	Inhibition	22.4 μM		HEK 293	[72]
		1B3	Fluorescein-methotrexate	Inhibition	365.1 nM		HEK 293	[68]
	Gallic acid	1B3	CCK-8	Inhibition	1.60 ± 0.60 μM		HEK 293	[64]
	Oleanolic acid	1B1	ES	Inhibition	28.5 μM		HEK 293	[72]
Red clover in soy Peanuts Chickpea	Biochanin A	1B1	DHEAS	Inhibition	11.3 ± 3.2 μM		HeLa	[74]
White mulberry	Mulberrin	2B1	ES	Inhibition	1.8 μM		HEK 293	[76]
Apple peel Olives	Betulinic acid	1B3	Fluorescein derivatives	Inhibition	368.2 nM		HEK 293	[68]

*ECG* epicatechin gallate, *EGCG* epigallocatechin gallate, *EC* epicatechin, *EGC* epicatechin-3-gallate, *β-PGG* 1,2,3,4,6-penta-*O*-galloyl-β-d-glucose

*ES* estrone sulphate, *DHEAS* dehydroepiandrosterone sulphate, *BSP* bromosulphophthalein, *CCK-8* cholecystokinin

Food such as pomegranate and olives contain antioxidants and other naturally occurring chemicals [64]. Studies have implicated food-derived chemicals interacting with OATP influx transporters [63, 68]. For instance, consumption of fruit juices such as grapefruit, apple and orange juice, can significantly reduce the oral bioavailability of drugs due to the inhibition of intestinal OATPs [69].

In most cases, the interactions of herb/food-derived chemicals with OATPs involve competitive inhibition of substrate transport, or allosteric inhibition due to altered transporter conformation. Other potential mechanisms include altered transporter expression, subcellular localisation or stability, but definitive evidence for such mechanisms is sparse. In general terms, changes in protein expression and/or rates of protein degradation occur over a relatively long timeframe, while functional modulations due to altered trafficking or interference with substrate binding are more rapid [70]. In this review, we discuss the relationship of the intake of both herb- and food-derived chemicals with the impairment of OATP function. The application of such information may prevent adverse effects of conventional drug therapies and improve treatment outcomes.

### Drug-herb interactions involving OATPs

A wide range of herbal compounds have been found to modulate the substrate uptake mediated by OATPs (Table 1). For example, kampo products have been used in Japan as traditional herbal medicines in treating inflammatory bowel disease, nausea, diarrhea and gastrointestinal tract disorders for over 1500 years. Kampo products consist of crude extracts from more than 98 sources, such as Bofutsushosan, Rhei Rhizoma, Perillae herba, Scuterallia Radix, Glyryrrhizae Radix, Moutan cortex, Paeoniae Radix, Tribuli Fructus, Saussurea Radix and Curcumae Rhizoma. Japanese physicians have also recommended these products to cancer patients as supplements to ongoing chemotherapy and radiotherapy [80].

Kampo extracts contain a range of bioactive chemicals that have been shown to modulate OATP transport function in cells that over-express these transporters [67, 71, 72, 81]. Thus, complex polyhydroxylated multi-ring systems like the catechins Epigallocatechin gallate (EGCG) and epicatechin gallate (ECG), flavonoids such as scutellarin and baicalin, as well as saponins like glycyrrhizic acid, have been reported to inhibit the transport function of OATP1B1 and 2B1 (Table 1). In contrast, the glycosylated flavonoid rutin increased the substrate transport via OATP1B1 and OATP2B1 in over-expressing HEK 293 and HeLa cells [71, 74]. Because these transporters are widely implicated in the cellular influx of drugs like fexofenadine, glibenclamide, statins and β-adrenergic antagonists, there is a considerable potential for pharmacokinetic interactions with Kampo-derived chemicals.

Baicalin and its aglycone baicalein are flavonoids that are active constituents of Chinese skullcap. The flavonoid scutellarin is enriched in breviscapine. Extracts from these natural sources have been found to impair the transport function of OATPs in the over-expressing cells [67, 72, 76]. Horny Goat Weed is a widely used Chinese medicine in Asian countries. It contains the prenylated flavanol glycoside icariin, which is used to treat osteoporosis and male sexual dysfunction. However, icariin has been found to modulate the transport function of OATP2B1, OATP1B1 and OATP1B3 [27, 63]. Milk thistle contains the flavonolignan silymarin—a polyhydroxylated ring-containing molecule, which potently inhibits estradiol-17β-glucuronide uptake via OATP1B1 and 1B3, as well as ES uptake via OATP2B1 [75]. There are other herbal extracts containing chemicals that can modulate the substrate uptake mediated via OATPs. The triterpenoid saponins astragaloside and ginsenoside Rc found in Radix astragali and Panax ginseng extracts, the naphthodianthrone 2-O-galloyl-hyperin enriched in Pyrola incarnate Fisch as well as the glycosylated flavonoid epimedin C present in Herba epimedii, have all been shown to inhibitors of OATP1B1 with moderate potency [72].

The capacity of molecules to modulate the rate of transporter-mediated substrate influx into cells has been reported but is under-explored. Again, the underpinning molecular mechanisms could be either substrate-dependent or substrate-independent. Chemicals that enhance the translocation of OATPs to the plasma membrane could activate the influx of chemicals in a substrate-independent fashion. Amiodarone (a potent natural compound used in the treatment of cardiac arrhythmias) and rutin have been shown to increase OAT2B1-mediated substrate uptake via increased translocation to the plasma membrane [82, 83].

Molecules that bind directly to specific domains or regions within OATPs could elicit rapid changes in substrate uptake [83]. For example, the steroid progesterone has been shown to activate OATP2B1 function by promoting a conformational change in the substrate-binding site [70]. Similar allosteric interactions have also been indicated to activate CYP monooxygenases and increase the rate of substrate oxidation [84].

### Drug-food interactions involving OATPs

Therapeutic complications due to drug-food interactions involving OATPs have also been demonstrated (Table 1). Grapefruit and apple juices contain a range of flavonoids that have been found to impair the transport function of OATP1A2, 1B1, 1B3 and 2B1, some with IC_50_ and K_i_ values in a low micromolar range [67, 72, 77, 78]. In vivo studies in patients have suggested that co-administration of grapefruit, orange or apple juices decreased the systemic availability of fexofenadine by 65–75% and celiprolol by more than 80% [85, 86]. Such inhibitory effects of fruit juices are reversible as the removal of fruit juice restores OATP function [69]. Literature also reported that ingestion of fruit juices that contain high concentrations of naringin directly inhibits enteric OATP1A2 and decreases the oral bioavailability of fexofenadine [77, 85]. Accordingly, it has been suggested that the consumption of fruit juices should be avoided within 4 h of drug administration to minimise adverse effects.

Green and black teas are popular beverages that are widely consumed with potential health benefits. Catechins and polyhydroxylated flavonoids such as theaflavin, that are present in teas can impact on OATPs and reduce the systemic exposure to OATP drug substrates like rosuvastatin [87].

Other classes of natural compounds that are potent in modulating OATP activities, are present in vegetables and fruits widely used in health treatments. For instance, licorice contains the saponin glycyrrhizic acid, which has been in managing chronic hepatitis and gastric ulcers. Glycyrrhizic acid was reported to inhibit the cellular uptake of OATP substrates atorvastatin, fluvastatin and rosuvastatin [76]. Literature also indicated that chemically similar molecules like the triterpenoid saponins ursolic acid, oleanolic acid and betulinic acid, are potent OATP inhibitors [64, 68]. Ursolic acid is present in pomegranate with anti-mutagenic and anti-viral properties. It can inhibit the OATP1B1- and OATP1B3-mediated uptake of fluorescein-conjugated methotrexate analogues [88]. Similarly, biochanin A in peanuts and mulberrin in mulberries are flavonoids that have been found to inhibit OATP1B1 and OATP2B1, respectively, in a non-competitive manner [74, 76, 89]. Thus, food-derived chemicals, if ingested in enough amounts, have the potential to influence the safety and efficacy of co-administered drugs.

### Clinical significance of drug-herb/food interactions mediated through OATPs

There is limited clinical evidence available regarding drug-food/herb interactions via OATPs [70]. In healthy Chinese volunteers, the flavonoid quercetin present in apples and other fruits, significantly decreased the bioavailability of pravastatin (an OATP1B1 drug substrate), most likely by decreasing intestinal absorption [72]. Another clinical study examined the influence of fruit juices on the disposition of the antihistamine fexofenadine. The oral pharmacokinetics of fexofenadine was assessed in ten healthy individuals received grapefruit, orange or apple juice (1.2 L over 3 h) in a randomized 5-way crossover study. It was found that grapefruit, orange, and apple juices decreased the fexofenadine area under the plasma concentration–time curve (AUC), the maximal plasma drug concentration (C_max_), and the urinary excretion values to only 30–40% of control [85]. Another study found that grapefruit juice decreased the C_max_ of acebutolol (an OATP1A2 substrate) by 19%, although the AUC was essentially unchanged from control [81]. Similarly, the administration of orange juice was found to decrease the C_max_ and AUC of the β-adrenoceptor antagonist atenolol (a common substrate of OATP1A2 and 2B1) by 49% and 40%, respectively [73]. There are also pharmacokinetic studies reporting that green tea decreased the absorption of rosuvastatin in healthy volunteers due to the inhibition of intestinal OATP1A2- and OATP2B1-mediated drug uptake [87, 90]. Additionally, green tea has been shown to interfere with pharmacokinetics of nadolol in Japanese volunteers, possibly due to a decrease in absorption via influencing OATP1A2 and OATP2B1 [90, 91].

## Conclusions

OATP transporters are widely distributed in human tissues and mediate the influx of many drugs and endogenous substances. Food and herbs are important sources of nutrients with potential health benefits. Accordingly, the potential of drug-herb/food interactions due to concurrent use of food and herbal agents alongside conventional drugs is high, which may greatly impact on therapeutic outcomes and toxicities. Increased awareness of these interactions could inform precautions to avoid co-administration of herbal medicines or food with OATP drug substrates to improve clinical outcomes.

## Data Availability

All the data used to support the findings of this study are available from the corresponding author upon reasonable request.

## Abbreviations

AUC: Area under the plasma concentration–time curve
C_max_: Maximal plasma drug concentration
EC: Epicatechin
ECG: Epicatechin gallate
EGC: Epicatechin-3-gallate
EGCG: Epigallocatechin gallate
ES: Estrone-3-sulfate
OAT: Organic anion transporter
OATP: Organic anion transporting polypeptide
β-PGG: 1,2,3,4,6-penta-*O*-galloyl-β-d-glucose
SLCs: Solute Carrier transporters
